# The Eukaryotic Cell Originated in the Integration and Redistribution of Hyperstructures from Communities of Prokaryotic Cells Based on Molecular Complementarity

**DOI:** 10.3390/ijms10062611

**Published:** 2009-06-04

**Authors:** Vic Norris, Robert Root-Bernstein

**Affiliations:** 1 AMMIS Laboratory, EA 3829, University of Rouen, Mont Saint Aignan, 76821 France; E-Mail: victor.norris@univ-rouen.fr (V.N.); 2 Department of Physiology, 2174 BPS, Michigan State University, East Lansing, MI 48824, USA

**Keywords:** composome, intron, motility, molecular complementarity, hyperstructure, origin of life, evolution, cytoskeleton, mutualism, symbiosis, ecology, ecosystem

## Abstract

In the “ecosystems-first” approach to the origins of life, networks of non-covalent assemblies of molecules (composomes), rather than individual protocells, evolved under the constraints of molecular complementarity. Composomes evolved into the hyperstructures of modern bacteria. We extend the ecosystems-first approach to explain the origin of eukaryotic cells through the integration of mixed populations of bacteria. We suggest that mutualism and symbiosis resulted in cellular mergers entailing the loss of redundant hyperstructures, the uncoupling of transcription and translation, and the emergence of introns and multiple chromosomes. Molecular complementarity also facilitated integration of bacterial hyperstructures to perform cytoskeletal and movement functions.

## 1. Introduction

Several characteristics distinguish unicellular eukaryotes from prokaryotes: 1) eukaryotes tend to be more effectively motile than a comparable mass of prokaryotes; 2) eukaryotes have endomembranes, which many prokaryotes do not have; 3) eukaryotes have separated transcription from translation, while prokaryotes have not; 4) most prokaryotes have a single, circular genome, whereas eukaryotes have a genome distributed over several linear chromosomes (we will discuss exceptions below); and 5) eukaryotes utilize introns within transcribed genes, while prokaryotes do not (another point we will elaborate below). It would be easy to look at each of these features as something that eukaryotes *gained* during the course of evolution, but we would like to suggest that each of these features is actually the result of a process of *loss*. In order to understand our argument, it is necessary to think of evolution not as the elaboration of individual traits, but as selection for more and more efficient *ecologies*. Thus, we propose that eukaryotic species did not evolve from elaboration or complexification of individual prokaryotic species, but rather that i*ndividual* eukaryotic species evolved by integration and simplification from *communities* of diverse prokaryotes. By integrating a community of prokaryotes into a single cell, eukaryotes integrated many of the best features of that community, thereby localizing their functions. By taking prokaryote functions that were previously distributed and duplicated within the community, integration into single cells would have produced more energetically efficient, better buffered, and more robust systems. Integration of partial duplications, however, would have resulted in incomplete overlapping of some features, resulting in some functions being distributed within eukaryotic cells, including features such as multiple internal membranes; multiple (partial) genomes; both nuclear and cytoplasmic (mitochondrial) DNA; and genes only partially integrated and therefore characterized by having exons punctuated by introns.

The theory that we present here is a logical consequence of an “ecosystems first” theory of the origin of life that we have been developing over the last few years [1–3]. In essence our “ecosystems first” theory argues that what evolves are not individual species but integrated systems [4–6]. A system is defined as being composed of components the possible combinations of which are greater than one but significantly less than the purely probabilistic or combinatorial possibilities. A system, in other words, is capable of varying its constituents and organization in response to its environment, but in order to retain its organization, is limited in the extent to which it can vary. An ecosystem is one that is based in the natural physicochemical and/or biological ecology of its environment and evolves with it.

We have proposed, unlike previous theories of the proteinoid or RNA-based theories of the origins of life, that what gave rise to living systems was the evolution of a chemically diverse environment in which every possible prebiotic compound that could be produced was produced in every possible chemical environment. Thus, prebiotic ecologies were diverse and would have been characterized not only by the presence of RNAs and amino acids or short peptides, but also polysaccharides, lipids, porphyrins, etc. Selection among this diverse ecology was carried out in the first instance by molecular complementarity. Those molecules that could bind to each other did so, resulting in complexes that were stabilized against degradative processes, survived for longer periods of time, and were therefore able to take place in further chemical combinations and reactions. Molecularly complementary complexes also have emergent properties not found in their constituents, so molecular complexes provided novelties upon which further evolution could operate. The result of this process of selection by molecular complementarity would have been the emergence of a new chemical ecology of compositionally diverse chemical complexes, which we call “composomes” [1,7]. Such composomes (which include coacervates, micelles, vesicles, etc.) have been created in the laboratory and been shown to self-organize, carry out a variety of chemical reactions, and to replicate [1]. Eventually, the diversity produced by composomal evolution would have resulted in spatially distributed networks of composomal “species” that were able to catalyze the production of each other’s constituents. In other words, composomal catalytic reactions would have resulted in synthetic hypercycles. Such distributed hypercyles of composomal reactions would have been more efficient than the random reactions producing the constituents of these composomes from a purely chemical ecology, but also inherently inefficient compared with a structurally integrated complex that could itself produce all of the compounds required for its own self-replication. Through further selection for molecularly complementary components, distributed networks or hypercycles eventually became integrated into the more complex organizations of localized chemical reactions (autocatalytic cycles) that we recognize as protocells and viruses (including the predecessors of bacteriophage). A key point is that we conceive of evolutionary processes as not only branching, but integrating, so that the proper model is not a tree, but a network [4]. In consequence, we do not believe that there was a single ancestral species or protocell, but that, just as there are many possible composomal species, life emerged multiple times through the integration of a variety of different composomal aggregates. Ecologies give rise to ecologies.

The key principles of our approach to the origins of life are that everything that can happen does happen, and that the resulting diversity is continuously pruned by molecular complementarity, which selects from this diversity those constituents able to stabilize and interact functionally with each other [3]. The result of such a scenario is that living systems are, by necessity, highly integrated at a molecular level, this integration being reflected in the high degree of molecular complementarity between the constituents. This molecular complementarity is often exhibited by stable modularity. Integration based on molecular complementarity and modularity is clearly evident at every level of organization in all cells, but perhaps most clearly in hyperstructures such as nucleoli, DNA-histone complexes, enzyme assemblies responsible for substrate channeling, actin-based structures, ligand-receptor-second-messenger systems, etc.

Our purpose in this paper is to describe how the principles we have previously developed can be applied to explaining how eukaryotic cells evolved from prokaryotes. Our basic hypothesis is that just as the evolution of diverse chemical ecologies gave rise, through the pruning of molecular complementarity, to diverse composomal ecologies, and the evolution of diverse composomal ecologies gave rise, through the pruning of molecular complementarity, to diverse prokaryotic cells, so a diverse prokaryotic ecology gave rise, through the pruning of molecular complementarity, to a new eukaryotic ecology. We propose that the emergence of prokaryotic cells resulted in tremendous diversification of prokaryotic species that evolved to take advantage of new niches. This diversification resulted in specialization, and also in some prokaryotic species interacting mutualistically and symbiotically with others. Since such interactions were spatially distributed, however, these mutualistic and symbiotic interactions were subject to dissociation and interference, creating selection pressures for spatial integration. Probably the first instantiation of such spatial integration would have been selection for mutualistic prokaryotes that attracted each other by chemical messages so that they co-localized. Among these, some not only attracted, but also bound to one another through complementarity between their cell surface molecules. Such mutualistic selection would have required compatible (i.e. complementary) receptors and cell-surface adherence molecules (perhaps the ancestors of such modern molecules). Csaba [8,9] has proposed that such complementarity evolved through a process that he calls “hormonal imprinting” by which small molecules select among random proteins for those that can bind them. This selection “imprints” the cells so that they produce higher levels of such hormone-binding proteins. Dwyer [10,11] in contrast, has proposed that small molecule homo- or self-complementarity formed the basis of receptor evolution, so that receptors often contain in their binding regions copies of their own ligands. Such receptors would be complementary not only to their ligands, but potentially to each other. Root-Bernstein [12] has extended Dwyer’s theory to incorporate heterocomplementarity as well, so that small molecules that are complementary to each other may provide the material from which each other’s receptors evolve. For example, insulin is homocomplementary as well as heterocomplementary to glucagon, and copies of insulin are found in the binding regions of both the insulin and glucagon receptors. Similarly, insulin binds glucose, and multiple copies of insulin make up the glucose transporter transport cores. In all of the theories just summarized, selection for small molecule binding would result in prokaryotic receptors responsive to cellular messages and also potentially complementary to each other. Koch *et al*. [13] and Steinberg [14] have developed mathematical models showing that the non-random binding between cells that would result from inter-cellular complementarity produces just the kinds of organized, dynamic, multiple cell-type clusters that we are proposing here as the precursors of eukaryotes.

Eventually, the co-localization of two or more species of mutualistic or symbiotic prokaryotes that could physically interact with each other would have led to the possibility of merging cell membranes and walls, resulting in meta-cells. Meta-cells would have had many benefits over independent cells in terms of energy efficiency and in preventing the loss of mutualistic and symbiotic messages and shared substrates and products through environmental diffusion. On the other hand, meta-cells would also have had certain drawbacks, including difficulties regulating very large structures, unnecessary duplication of cellular machinery, and probably some conflicts of control and organization. As nature often does in such instances, pruning would have occurred, eliminating as much redundancy as possible, and thereby reducing the size of such meta-cells to produce more efficient, integrated cells. Such spatial integration would have required a compatibility between the molecular hyperstructures constituting the cells in each of the mutualistic and symbiotic species of prokaryote that contributed to this new meta-cell; achieving this structural and functional compatibility within a single even larger unit created what we now recognize as a eukaryotic cell. We do not believe that this happened just once, but probably many times so that eukaryotes have multiple origins, though probably within a limited geological time-frame. We are therefore going beyond the “revolution” that Goldenfeld and Woese [15,16] proposed in which sharing of genes across species must make us rethink biology, to a model in which both genes AND entire non-genetic structures, such as membranes, cytoskeletal components, etc. are also shared.

There are many consequences of the model we have briefly introduced above, each of which would require significant space to draw out and justify. In this paper, we will focus mainly on the integration of structural cellular machinery and the emergence of motile functions, leaving the bulk of discussion of intron-exon, nuclear encapsulation of replication/transcription, and similar issues for other papers.

## 2. Colony Model for the Creation of the Eukaryotic Cell

It is now believed that the adhesion of genetically distinct strains is widespread and there is evidence for coaggregation amongst bacteria isolated from biofilms in the gut, urogenital tract, dental plaque and water supplies [17]. In a community of freshwater bacteria, *Blastomonas natatoria* coaggregated specifically with all 18 other bacteria. Coaggregation brings more than just resistance to hydrodynamic and other forces. There are over 500 taxa of oral bacteria and, in an *in vitro* study of dental plaque, the coaggregating partnership of *Streptococcus oralis* and *Actinomyces naeslundi*i formed a nutritionally beneficial, mutualistic relationship that allowed each to grow where neither grew alone [17]. We assume that similar coaggregates of prokaryotes emerged early in prokaryotic evolution and became widespread.

What, then, would have been the advantages of being a single proto-eukaryotic cell rather than a mixed colony of bacteria?

### 2.1. Stable composition

If the success of a mixed, mutualistic, synergistic, and/or symbiotic colony depends on a particular composition in terms of different prokaryotic species, this success is imperilled if the constituent bacteria are independently dispersed or if one species is subject to environmental stresses that another is not. Hence there would have been an advantage for symbiotic colonies of bacteria to integrate their functions into non-dissociable forms.

### 2.2. Energy efficiency

Distributing functions among discrete prokaryotic species in a mixed colony requires that shared resources or regulatory messages diffuse between cells. Such diffusion is susceptible to loss or weakening of resource or message; interference from poisons or environmental noise; and hijacking and use by non-mutualist and non-symbiont species (e.g. parasites and pathogens). Integration of symbiont functions within a single cell provides much higher efficiency of function and less energy to obtain the same outcomes.

### 2.3. Increased mobility

Bacteria move in liquids via a wide variety of mechanisms including chemotaxis, passive diffusion and growth (i.e. spreading through replication). Even though bacterial chemotaxis is very effective, heterogeneity is ever-present. First, there is heterogeneity even within the same species. In a homogeneous environment, steady state tumbling frequencies and adaptation times vary in genetically identical bacteria [18]. Moreover, bacteria change their environment chemically and physically. When *E. coli* migrated from one end of a capillary tube towards galactose, the population formed two bands with the first band consuming all the oxygen to oxidize some of the galactose and the second band using the residual galactose anaerobically [19]. Hydrodynamic disturbances caused by bacteria proximity were found to increase with increasing cell density, leading to an increase in the diffusion coefficient [20]. Second, even if the movements of millions of individual bacteria of in a single species in some conditions were to be collective, in other species and in other conditions, these movements may be uncoordinated. Clearly, the movements of multiple symbiotic species will be even more susceptible to randomness. The movements can be slow, wasteful (trails of bacteria left behind) and difficult in 3D in liquid. This problem becomes multiplied exponentially if multiple species of mutualistic or symbiotic prokaryotes need to move or grow synchronously and in tandem, but diffuse randomly. Integrating multiple species of prokaryotes into a single eukaryotic cell takes care of the diffusion problem, but creates a movement problem of its own – namely how is this cell to move, especially given its increased size and therefore hydrodynamic drag? So larger, integrated cells required the evolution of movement structures, and in particular, the fusion of the bacterial cytoskeletal hyperstructures was needed to form the core of cilia and an actin cytoskeleton and hence provide motility and coordination of motility.

### 2.4. Pooled resources

Mixed colonies of symbiotic prokaryotes would be susceptible to death if individual component species succumbed to environmental stresses. Lysis of bacteria to provide nutrients for others occurs frequently [21–26], but is probably wasteful and hard to coordinate. Moreover, colonies have different sizes and can break up into their individuals. Evolving a larger eukaryotic cell that integrates mutualistic and symbiotic functions and perhaps duplicates some of these for backup (redundancy), has a constant composition (as compared with the varying constituents of a mixed colony) and has a relatively constant size, would have maximised the use of reserves in difficult conditions.

### 2.5. Increased phenotypic range

The phenotypic range of the collection of individual prokaryotes within prokaryotes may be less – or at least less exploitable – than that of the single proto-eukaryotic cell which contained much of their DNA and many of their hyperstructures. Successful combinations of prokaryotes that became fully integrated as eukaryotic cells would have been more stable and therefore have a much higher probability of reproducing accurately and surviving for longer periods of time than loosely organized mixed colonies in which every component species can vary and is under individual selection pressures.

## 3. Achieving the Transition from Colony to Single Proto-Eukaryotic Cell

Creating the eukaryotic cell from a mat, biofilm or colony of diverse species of bacteria entailed surrendering individual control and weakening individual identity so as to gain coordination of responses to the environment and cell cycle synchrony. This may have been achieved by the redistribution of hyperstructures from multiple cell types into a single cell type. This redistribution could have occurred in two ways: the combining of cytoskeletal hyperstructures and the loss of hyperstructures depending on coupled transcription and translation. This redistribution may have been enabled by the lysis that many species of bacteria undergo that leads to their contents being released and taken up by others. It could also have been enabled by widespread transduction by bacteriophage. The success of redistribution depended on the compatibility of the constituents of hyperstructures across the species. We hypothesize that this compatibility existed throughout the prokaryote ecology due to the fact that the evolution of these constituents was tightly constrained by molecular complementarity from the earliest steps of evolution. In fact, the very ability of bacteriophage to infect and replicate within bacterial species is predicated on the complementarity between both the proteins that recognize specific bacterial species and their ability to hijack the genetic machinery of the infected cell. Bacterial lysis, which is common in modern bacteria, is divided into three classes [21].

### 3.1. Autolysis in genetic transformation

Competence for genetic transformation is induced in the early exponential phase in bacteria such as *Streptococcus pneumoniae* by a quorum sensing mechanism involving a peptide hormone, and cells in the competent state can take up exogenous DNA. Competent cells are more prone to autolyse to release DNA. Competence, stress responses and autolysis are related. Competent cells are also able to lyse competence-deficient cells (and release their DNA) during cocultivation in the phenomenon of ‘allolysis’ [22] and induced lysis of genetically identical cells is known as ‘fratricide’.

### 3.2. Developmental lysis

Developmental lysis during starvation in *Myxococcus xanthus* involves the autolysis of most of the cells (up to 90%) of the initial population – within the first 72 h of development of the fruiting body. It is assumed that lysis of these cells is a regulated phenomenon of ‘programmed cell death’ and that the nutrients released by the lysed cells feed the sporulating cells [23]. A similar phenomenon of ‘cannibalism’ occurs in the early sporulation stage of *Bacillus subtilis* (although this species does not form a fruiting body) in which some starving cells secrete factors to kill and lyse other *B. subtilis* cells that have not developed immunity to these compounds [24]. In the case of *Epulopiscium sp.* type B, the mother cell undergoes a programmed cell death that is believed to allow for the timely release of resources accumulated in the mother cell to provide nutrients to populations of these intestinal microbes *AND* their host, the surgeonfish [25].

### 3.3. Lysis of prey cells

Both actinomycetes and myxobacteria produce bacteriolytic exo-enzymes (as well as immunity factors for self-protection). For example, swarms of thousands of myxobacteria attack and lyse the many kinds of bacteria on which they feed using a wide range of extracellular lytic agents including antibiotics, bacteriocins, lipases, proteases and cell wall hydrolases [26]. A very different bacterium, *Yersinia pestis*, can produce a lysozyme to use against *Escherichia coli* and other *Yersinia* species [21].

Many species of modern bacteria can survive without an effective peptidoglycan layer as somewhat amorphous l-forms. Often these l-forms are unstable and, in the absence of penicillin, revert to being bacteria with apparently normal peptidoglycan. This bacterial property leads to one possible scenario for the origins of eukaryotes, which is that l-forms of prokaryotes integrated either with each other or within a prokaryote producing normal peptidoglycans. So as to prevent bacteria reforming in the early eukaryotic cell, we expect levels of lysozyme and other lytic agents would have been high in the proto-cytoplasm. Alternatively, prokaryotes that transiently went through an l-form phase could have integrated to produce a single protective peptidoglycan layer that surrounded the novel, integrated form without high levels of lytic compounds present. In either case, the ability to transition between protected and unprotected forms may have been a critical trait for the origins of proto-eukaryotic forms.

## 4. Bacteriophage and Plasmids

Another essential feature of the evolution of eukaryotes from prokaryotes was gene transfer between prokaryotic forms carried out by bacteriophage and plasmids. Goldenfeld and Woese have presented a strong case that such horizontal gene transfer was a critical feature of the origins of life [15,16], and one that substantially complicates the ability to formulate evolution as a divergent tree. Rather, functional modules were very likely transferred between cells by horizontal gene transfer. Selection for functionally efficient modules would have occurred, mediated as in previous stages of evolution, by compatibility with existing modules. This compatibility would have been mediated by molecular complementarity of the modules. Thus, viruses, bacteriophage, and plasmids could have been effective means of swapping elements to create novel combinations of functional modules.

We argue that viruses and bacteriophage evolved along with or even earlier than bacteria themselves and diversified by swapping modules among themselves as well as with bacteria as has been shown for the temperate and virulent bacteriophage of enteric bacteria using genetic and heteroduplex approaches [27,28]. For example, SopE, an effector protein in *Salmonella typhimurium* infections, is carried on a temperate bacteriophage [29]. It has been estimated that there are over 10^30^ bacteria [30] and over 10^31^ bacteriophage in the world. Hence, a considerable fraction of chromosomal DNA is carried by bacteriophage at any one time and transduction is of major importance in exchanging genetic material. It has been proposed that bacteria and bacteriophage form a single super-organism [31], or in our terms, an ecology. We have proposed that dynamic assemblies of bacteriophage replicating within bacteria constitute non-equilibrium hyperstructures whilst individual bacteriophage constitute what are effectively equilibrium structures that preserve their precious contents in a range of environments hostile to growing bacteria [2]. We have further proposed that this super-organism adapts and evolves via exchanges and alterations of hyperstructures [2]. The eukaryotic cell is one of the results of this evolution of the super-organism and it is therefore not surprising to find phage proteins playing key roles in the eukaryotic cell. For example, the mitochondrial RNA polymerase in *Saccharomyces cerevisiae* consists of a 145 kDa subunit with polymerising activity that resembles the RNA polymerase of the bacteriophage T7 and a 45 kDa specificity factor that resembles a bacterial sigma factor. Indeed, Forterre and others have proposed that DNA and the eukaryotic nucleus itself may have originated from infection of protocells with double-stranded DNA encoded viruses [28], an idea that is fully compatible with our hypothesis. Finally, we note that large plasmids, possibly of viral origin, are present in *Bacillus anthracis* and *Bacillus thuringiensis* that encode proteins believed to be involved in their segregation and have some structural and functional similarities with eukaryotic tubulin [32].

## 5. Types of Hyperstructure Involved

### 5.1. Cytoskeletal hyperstructures

But where did the hyperstructures that characterize eukaryotes evolve from? We believe that they are elaborations and integrations of prokaryotic hyperstructures. After many years of disbelief, it is now generally acknowledged that bacteria contain organized protoplasmic hyperstructures equivalent to those found in eukaryotes [33–35]. Significantly for our hypothesis that eukaryotes evolved by structural *loss* from more diverse prokaryotes, it appears that there are more cytoskeletal structures of greater diversity in bacteria than in eukaryotes [36]. We therefore propose that the loss of the envelopes around certain bacteria in mixed-species colonies allowed their cytoskeletal hyperstructures to combine, and that this combining was accompanied by loss of redundant and extraneous structures.

For example, the bacterial protein, FtsZ, has a structural homology to tubulin [37] and, *in vitro*, forms a wide variety of polymeric structures depending on the presence and concentrations of lipids, divalent ions and GTP [38]. Like eukaryotic tubulin, FtsZ also assembles into structure that plays an important role in cell division. Like its eukaryotic counterpart, the microtubule, the Z-ring is a highly dynamic structure with a turnover time for an FtsZ monomer within the ring of 10–30 s [39]. Intriguingly, production in *E. coli* of S100B, a human protein that undergoes a calcium-dependent conformational change to bind to tubulin, results in it colocalizing with FtsZ and inhibiting division [40]. In chloroplasts, which are related to cyanobacteria, FtsZ exists in the form of both a network and a ring at the site of division [41]. These observations clearly suggest that FtsZ could have been a molecular ancestor of eukaryotic tubulin.

A range of actin-like proteins also exist in bacteria (for references see [42]). Early evidence for a bacterial actin (for references see [43]), including sequence analysis [44], became difficult to ignore when actin-like filaments of MreB were discovered in *B. subtilis* [33]. The crystal structure of MreB was subsequently shown to resemble that of actin [45]; filaments formed by MreB have a short pitch (0.73 ± 0.12 mm) and assemble around the middle of the cell whilst those formed by Mbl, a structurally related bacterial protein, have a longer pitch (1.7 ± 0.28 mm) and cross the entire cell [33]. Thus, either or both MreB and Mbl may have provided the ancestral forms from which eukaryotic actins evolved.

### 5.2. Transertion hyperstructures

In bacteria, the coupling between transcription, translation and insertion of the nascent proteins into and through membrane – *transertion* – is the rule rather than the exception. This coupling is believed to prevent the formation of RNA-DNA hybrids [46]. It is also believed to generate large hyperstructures. When, for example, the *lac* operon is induced in an exponentially growing culture of *E. coli* (where it is present in more than one copy), the numbers of transcripts of *lacZ* per cell are 32 full length, 32 decaying and 38 nascent [47]. If the short half-life of most mRNA is discounted, the nascent transcripts would then be translated by 310 ribosomes. The same operon contains the *lacY* and *lacA* genes that can also be co-transcribed and translated. The result should be the formation of a hyperstructure comprising the *lac* genes dynamically attached to tens of nascent mRNAs, and to hundreds of ribosomes and the nascent enzymes. Notably, eukaryotic cells do not have the ability to produce such huge transertion hyperstructures, yet another instance of evolution by “loss” rather than gain.

### 5.3. Transembly hyperstructures

A *transembly* hyperstructure is created by the coupling between the processes of transcription, translation and assembly of the products into a structure. The equivalent of the eukaryotic nucleolus has long been proposed as existing in bacteria. In such a nucleolar/ribosomal hyperstructure rRNA genes would be transcribed and ribosomal proteins would assemble onto the nascent rRNA so bringing together genes encoding rRNA and ribosomal proteins, nascent RNA, and possibly nascent ribosomal proteins and their genes [48,49]. Evidence that such a ribosomal hyperstructure might exist in *E. coli* has been obtained by fluorescence studies *in vivo* of RNA polymerase tagged with green fluorescent protein [50]; these reveal that the distribution of RNA polymerase into transcription foci under rapid growth conditions corresponds to that expected from RNA polymerase recruitment to a nucleolar hyperstructure to transcribe actively several of the 7 sets of rRNA genes present in the chromosome (the authors claim one to three distinct nucleolar hyperstructures per nucleoid). In a search for such a hyperstructure in *B. subtilis* based on the different criterion of localization of *spo0J* and *rrn* genes, it was found that only those 7 *rrn* genes near the origin are colocalized in transcription foci whilst *rrn* genes further away are not colocalized [51]. Thus, once again, the components of eukaryotic hyperstructures such as the nucleolus can reasonably be hypothesized to have existed in at least some prokaryotes and could have evolved from these precursors.

## 6. Molecular Complementarity in the Context of Hyperstructures

The ribosome is a paradigm for the extent to which the evolution of the constituents of a structure is constrained by their multiple and necessary interactions. In these multiple, essential interactions, the constrained evolution of hyperstructures resembles that of ribosomes. Put differently, the operation of molecular complementarity in the billions of years before the origin of the eukaryotic cells meant that the interaction between several types of macromolecules within a single hyperstructure tightly constrained the evolution of these macromolecules. This meant that these macromolecules could continue to interact with their homologues produced by other bacteria to form new hyperstructures. Ecologies of organisms can therefore be said to impose constraints on variations across species that also promote modular compatibility and swapping among these organisms.

Molecular complementarity is the basis of both the constraint on variation and on the promotion of modular compatibility. A large body of evidence indicates that the division hyperstructure is held together by an intricate network of complementary molecular interactions between all of its constituents. The division hyperstructure in *E. coli* contains, at different times, combinations of the following proteins: FtsZ, FtsA, ZipA, ZapA, FtsE/X, FtsK, FtsQ, FtsL/B, FtsW, PBP3, FtsN and AmiC. FtsZ assembly into filaments is mediated by accessory proteins reminiscent of the way that Microtubule Associated Proteins control tubulin assembly into microtubules; these accessory proteins include FtsA, ZipA and ZapA of which ZipA is considered to best resemble typical MAPs [52]. The highly conserved ATPase, FtsA, assembles on the FtsZ ring to anchor it to the membrane and to determine its dynamics [39,53–55]. After formation of a stable Z-ring, the division hyperstructure is completed by the arrival of the late localizing proteins [56,57] such as FtsK, which is important for ensuring that the chromosome is not bisected by the division septum [58–63], FtsI, a transpeptidase important for building the cross wall [64,65], and the amidase AmiC which plays a role in coupling constriction of the outer membrane and the peptidoglycan layer to the cytoplasmic membrane in *E. coli* [66]. Preventing FtsZ assembly at aberrant locations is critical to maintaining the fidelity of cell division. The Min proteins are one means by which bacteria prevent non-productive division events at cell poles [67]. In *E. coli*, the Min system consists of three proteins, MinC, MinD (which resembles eukaryotic dynamin), and MinE [68]. MinC, is believed to inhibit the assembly of Z-rings by binding to FtsZ polymers and inducing displacement of FtsA or possibly by preventing FtsA binding to FtsZ polymers (for references see [52]). Another means of preventing aberrant division involves the DNA binding proteins SlmA in *E. coli* [69] and Noc (from Nucleoid Occlusion) in *B. subtilis* [70] which inhibit FtsZ assembly over the nucleoid. Other likely constituents of the hyperstructure include GroEL, which depends on FtsZ for its presence at the division site [71] and the phospholipid synthases, which in *B. subtilis*, are localized to the septal membranes in an FtsZ-dependent manner [72].

A similarly complex set of molecularly complementary interactions is evident in the system of molecules that determine cell structure. Actin-like MreB is an important determinant of cell shape in rod-shaped *E. coli* and *B. subtilis* as well as in the crescent-shaped *C. crescentus* where it is reported to organise a PBP2 complex involved in peptidoglycan synthesis and cell elongation into a band-like structure [73]; this complex is believed to contain PBP1a, PBP2a, PBP2b and PBP3a and possibly other enzymes responsible for peptidoglycan synthesis. Moreover, in *C. crescentus*, MreB also appears to form part of a kinetochore-like complex that specifically segregates the replication origin region of the chromosome [74]. In *B. subtilis*, where there are several MreB-like proteins, MreBH interacts with the autolysin LytE (a putative endopeptidase) to coordinate cell wall hydrolysis with cylinder elongation [75]. In *E. coli*, MreB interacts, physically and functionally with topoisomerase IV which mediates the resolution of topological linkages between replicated daughter chromosomes during chromosome segregation [76] whilst during cell division the MreB cytoskeletal ring also contains the MreC, MreD, Pbp2 and RodA proteins [77].

The importance of such complex systems of molecularly complementary interactions in evolutionary terms is that they demonstrate that it is not possible to view evolution as merely an accumulation of random mutations: systems such as hyperstructures impose their own natural selection on what variations are possible among the components. Any variation must remain functionally compatible with the modules already selected.

## 7. Motility via Cytoskeletal Hyperstructures

Mixing and matching compatible modules from diverse species of prokaryotes, and eliminating redundancies and extraneous modules, could have provided such important new eukaryotic hyperstructures as those required for cell structure and motility. In our hypothesis, lysis of bacteria within the protoeukaryote released cytoskeletal proteins that then interacted to produce dynamic structures that conferred motility. In support of this, both tubulin and actin homologues in modern bacteria are dynamic. In the case of the tubulin homologue, FtsZ forms dynamic helices in *E. coli* that have a dynamic activity on the scale of seconds along with slower oscillations of a minute or so [34]. Intriguingly, in mammalian cells, an FtsZ network can be colocalised with tubulin in the presence of drugs [78]. The significance of this is that even if the different ancestors of tubulin in prokaryotes did not assemble into copolymers, their networks may well have been colocalised and coordinated and therefore available for integration as hyperstructures in eukaryotes.

In the case of the actin homologues such as MreB, changes in the length and tension of cytoskeletal structures formed from two proteins, one of which is MreB, govern the motility of the wall-less, spiral-shaped bacterium *Spiroplasma melliferum* [79,80]. The filamentation of MreB is ATP-dependent [45] and the rate of extension of the growing end of filaments is similar to that of actin (0.1 micron/s), generating a potential poleward or centerward pushing velocity at 0.24 micron/min for MreB or Mbl, respectively [81]. Mbl, another actin homologue, is involved in maintenance of the cell wall In *B. subtilis*. A banded pattern is made along the long axis of the cell by labeled vancomycin binding to sites of peptidoglycan assembly, and this pattern depends on Mbl [82]. Turnover occurs along the length of the helical Mbl filaments, which have no obvious polarity and which appear to draw on a cytoplasmic pool that contains oligomers; the filaments are very dynamic and, when labeled and photobleached, have a recovery half-time of about eight minutes [83]. The helical pitch of the filaments in cells of various sizes and at different growth rates remains relatively constant. Since they move but do not have flagella it is thought that the dynamics of filamentous structures play a role in its motility. In particular, *Spiroplasma* bacteria propel themselves through viscous fluids by sending kinks of opposite handedness down their helical body. Their helical bacterial pitch angle is optimized for maximal speed and efficiency [80]. Such hyperstructures could have provided the material upon which eukaryotic hyperstructures were integrated.

## 8. Uncoupling Transcription, Translation, Insertion and Assembly

Sheer size would have become a problem for a meta-cell composed of multiple prokaryotic forms. One of the factors limiting the size of prokaryotes is the biophysical constraints of diffusion. When a gene is read directly into mRNA and translated at the transcription site, the protein products diffuse away from the gene. The rate of protein diffusion therefore limits the rate and extent to which proteins can reach targets such as the cell membrane, act as regulators or promoters on other genes, form complexes (such as ribosomes), etc. Muller-Hill [84], Kepes [85,86] and Jackson [87,88] have therefore argued that prokaryotic genome organization was optimized by selection to bring interacting genes and gene products together by folding. Such folding minimized the distances between genes producing interactive and regulatory proteins so that rates of diffusion were not major factors in gene regulation and cell metabolism. For the same biophysical and energetic reasons, gene products were selected for their ability to form functional complexes such as those that govern channelling [89]. Integrating two or more prokaryotic genomes into a single meta-cell would, however, have undone much of what evolutionary processes had previously optimized. In return for integration, the size of the cell would have increased so that diffusion of gene products would have become a serious limitation on cell function. (Recall, however, that this integration would also have optimized inter-cellular communication and symbiotic sharing of metabolites and messages so that two different optimization strategies would have been in competition.) Moreover, integration would have resulted in unnecessary duplication of many critical genes. The obvious solution, which has been observed in many instances, was for evolutionary processes to prune out unnecessary gene duplications from the multiple genomes. We propose that one result of this pruning was individual chromosomes. Such chromosomes would have retained the optimized folding of individual groupings of genes and gene products but would also have permitted crossing over and cis-trans interactions to occur between similar (but not identical) regions of these emerging chromosomes resulting in integration of similar genes, or different genes regulated by similar promoters and repressors.

When, then, did multiple, linear chromosomes evolve? As noted in our introduction, some exceptional prokaryotes have multiple chromosomes and plasmids, and some of these may be linear [90–93]. Evidence suggests that the same factors that promoted the diversification of multiple, linear chromosomes in eukaryotes were also at work in these exceptional prokaryotes and that, in some cases, multiple and linear chromosomes and plasmids in prokaryotes may be the result of horizontal gene transfer from eukaryotic hosts via viruses. One of the key observations is that when multiple chromosomes are present, there tends to be one large one that is highly stable and contains the vast majority of essential prokaryotic genes while highly variable genes that may be of value in variable environmental conditions are often found in the smaller chromosomes and plasmids [90]. Just as we have suggested that limitations on functional genome size and organization may, for physicochemical reasons, have required the evolution of multiple chromosomes in eukaryotes, the same factors have been postulated by Slater, *et al*. [93] to have led to multiple chromosomes in some prokaryotes: “The advantage of multiple chromosomes is unclear, but we speculate that they may permit further accumulation of genes when the primary replicon cannot support further chromosome enlargement. Within the *Rhizobiaceae*, different species appear to handle gene accumulation in different ways. *Bradyrhizobium* and *Mesorhizobium* species have very large chromosomes with few, if any, relatively small plasmids. In contrast, *Agrobacterium* and *Rhizobium* strains have multiple chromosomes or large replicons that show gene accumulation, as well as anywhere from one to six plasmids. These differences may suggest that chromosomal origins have differing abilities to replicate molecules larger than about 5 or 6 Mbp, with multiple chromosomes providing an alternative reservoir for newly acquired DNA.” Moreno [91] has further suggested that genome size may vary according to the environment in which the prokaryote is found. Prokaryotes that are free-living (say in soil, water, manure, etc.) may require larger numbers of genes divided into diverse genomes and equipped with adaptive flexibility through horizontal gene transfer mechanisms (e.g. plasmids) in order to survive environmental changes, whereas prokaryotes that live within eukaryotic cells may be able to eliminate many house-keeping genes, relying instead on their host, and therefore evolve smaller, single chromosomes.

Linearization of chromosomes may be due to the horizontal gene transfer mechanisms just referred to. In particular, there is some evidence that linearization of circular DNA (genomes or plasmids) can be achieved by the integration of a linear phage genome into circular DNA molecules [92]. Unlike circular chromosomes and plasmids, linear chromosomes and plasmids require some means to protect their free ends from exonucleases. Notably, the mechanisms that are found in prokaryotic linear chromosomes and plasmids are shared with both viral and eukaryotic linear chromosomes, and include hairpin sequences at the ends of the chromosomes, invertrons (in which the ends are covalently bound to proteins), and palindromic sequences that bind pairs of linear sequences to each other [90]. The fact that the same types of mechanisms are found in viruses, prokaryotes and eukaryotes either argues for a very early common ancestor or significant horizontal transfer between them. In the former case, the molecular machinery required for multiple, linear chromosomes in eukaryotes was invented but not fully utilized by prokaryotes; while in the latter case, it is possible that prokaryotes may have taken up the innovation of multiple, linearized chromosomes only after eukaryotes evolved and shared those innovations with them through horizontal gene transfer mechanisms [94].

An important consequence of integrating diverse prokaryotic genomes would also have been the production of introns. Crossing over could have produced “mixed” genes attached to a single regulatory region but several active regions. Such integrated genes would have retained some of the genetic material from their original genomes resulting in exon-intron-exon motifs. If this scenario is accurate, then introns may represent partial gene (exon) sequences carried over from the original prokaryotes and so provide a means to identify the species origins of different genes brought together by the integration we are proposing here.

Again, we propose that the materials necessary for intron formation in eukaryotes were adapted from existing structures within prokaryotes and that integration of multiple prokaryotes into a single cell necessitated their new function. It has been discovered that many (perhaps even the majority) of prokaryotes contain *intron-like* sequences in their genomes (so-called Group I and Group II introns), but it is generally agreed that these sequences do not function as introns in prokaryotes, but rather as retroelements. Edgell *et al*. [95] and Rudi *et al*. [96] found that Group I introns have a “distribution pattern that resembles a pattern expected for a mobile element” such as retrotransposons and their distribution can best be explained not by selection for enhanced protein processing but by random horizontal gene transfer. In fact, these elements are never found within genes and retain their reverse transcriptase activity, “hence,” as Koonin [97] has argued, “formally, losing the intron status.” Similarly, Dai and Zimmerley [98], conclude that bacterial intron sequences are, “not present in conserved genes and are often located outside of genes… No introns have yet been identified in bacteria that are expected to function only in splicing (i.e. ORF-less introns or introns with degenerate ORFs); however, there are many examples of introns that resemble retroelements due to insertion into the wrong strand of a gene, insertion outside genes or insertions after a terminator structure.” Toor *et al*. [99] have therefore proposed that eukaryotic introns evolved from these retroelements in bacteria: “all currently known group II introns were derived from mobile bacterial group II introns. The catalytic RNA structures were proposed to have differentiated in bacteria as components of retroelements, followed by ORF loss in mitochondria and chloroplasts to form the numerous organellar ORF-less introns.” This hypothesis is consistent with Koonin’s [97] hypothesis that eukaryotic introns evolved through horizontal gene transfer of retroelements throughout the viral and bacterial kingdoms but were coopted into novel functions only in eukaryotes. Both the Toor-Hausner-Zimmerly and Koonin hypotheses are fully compatible with our proposal that eukaryotic metastructures and their novel properties evolved from horizontal gene transfer and integration of diverse bacterial components.

Incorporation of multiple prokaryotic functions into a single eukaryotic cell would, however, have resulted not only in integration, but selection for new functions such as the separation of transcription from translation. Uncoupling transcription and translation made it easier to combine mRNAs or parts of mRNAs from different bacteria by using splicing. Separate transcription-translation hyperstructures would have made such splicing difficult and would have limited the phenotype of the meta-cell to that of individual bacteria. In other words, introns and separation of transcription-translation needed one another. An additional reason for this separation of functions would have been the increased size of the eukaryotic cell. The same biophysical considerations, such as limiting diffusion to reduce feedback times and the rates at which products reached their destinations, that optimized gene and protein organization in prokaryotes would not have been able to function in significantly larger cells. Multiple genomes distributed in different parts of the cell could not have communicated fast enough to perform functional regulation. Gene products could not have diffused to their cellular destinations in a timely fashion. Thus compartmentalization of these functions evolved in conjunction with specialized distribution networks.

The most obvious form of compartmentalization was the separation of the genome from the cytosol by means of a nuclear membrane (most likely a residual of one of the prokaryotic contributors to the eukaryotic cell). The nucleus served to co-localize the genomes of the disparate prokaryotic contributors to the eukaryotic cell, optimizing genetic regulation and providing means to prune the redundancies out of the genomes. The nucleus also separated transcription from translation. Such separation was again necessary to optimize translation. Proteins in a significantly larger cell needed to be made at or near the sites at which they would be used. Localizing the translation apparatus in a stable structure such as the endoplasmic reticulum rather than having to recruit ribosomes from the cytosol by diffusion would have been much more efficient. Similarly, utilizing a “delivery track” made up of actins would have reduced the degrees of freedom required for diffusing protein products from three dimensions to one dimension (along the actin fiber) resulting in vastly increased rates of protein delivery.

In sum, as with the previous processes that we have discussed, integration resulted in increased efficiency of some aspects of transcription and translation, but led to other problems that needed to be optimized by pruning of the integrated functions. In this instance, the uncoupling of transcription, translation and insertion led to:

- The loss of bacterial transertion hyperstructures.
- The formation of cotranslational insertion hyperstructures and the fusion of these hyperstructures to give the endoplasmic reticulum.
- The formation of transcription hyperstructures and their fusion to give the nucleus.
- New, essentially eukaryotic, hyperstructures in which transcription, splicing and export are coupled and in which splicing made DNA from different bacteria compatible.

One of the unique predictions made from this scenario is that the different chromosomes that are found in primitive eukaryotes may retain a “molecular paleontology” of their origins, so that each chromosome may have significant homologies to parts of the genomes of different contributing prokaryotes. If this prediction is correct, it should be possible to trace to some extent the contributions of diverse prokaryotes to the evolution of eukaryotic cells.

## 9. Discussion

In sum, we are proposing a significant modification of the endosymbiont theory of eukaryotic origins developed by Margulis [100]. Margulis proposed that symbiosis led to some prokaryotes incorporating what we now recognize as either chloroplasts or mitochondria within themselves. Such endosymbioses were one-time events that occurred, according to Margulis’s scenario, between pairs of specific prokaryotes. All modern eukaryotes evolved from these origins. We propose an ecological origin of eukaryotes that resulted in distributed poly-phyletic evolution of eukaryotes. We propose that much broader forms of gene and hyperstructure sharing was occurring continuously within the prokaryotic ecology that preceded the evolution of eukaryotes. This sharing began as disseminated mixed colonies, mats or films of mutualistic prokaryotes. Sharing of genes and other hyperstructures was mediated in part by viruses and bacteriophage that infected and became synergistic with these mixed colonies, mats and films. Mutualism evolved into symbiosis as more and more integration occurred and interactions became more highly refined and efficient. Symbiosis, mediated by conserved molecular complementarities between diverse modules, eventually led to the emergence of meta-cells that incorporated not just proto-chloroplasts and proto-mitochondria, but much more diverse combinations of prokaryotic elements. These diverse elements led to the breakdown of single, circular genomes in favour of multiple, discrete, linear chromosomes in order to prune redundancy and promote cis- and trans-gene control. The intron-exon system of editable mRNAs is a consequence of this integration of diverse genomes, as is the dissociation of transcription from translation. At the same time, certain key bacterial hyperstructures were conserved and simply extended. These include the cytoskeletal and motility hyperstructures. Others, such as the ribosomal hyperstructure, were modified and extended.

The key concepts that we assume, and for which we have provided evidence here, are that all critical elements of eukaryotes have structural precedents in prokaryotes; that the relative complexity of eukaryotes can be explained by the merging of diverse prokaryotic elements or modules; that such integration of diverse elements and modules is a characteristic of ecologies and is made possible by shared molecular complementarities that were at work across all living systems; that integration is always associated with pruning of redundant or conflicting modules and systems, which in turn gives rise to novelties (such as dissociated transcription/translation and the intron-exon system); that replacing a diverse community of organisms with a single, integrated organism creates novel problems such as the need for motility and more highly defined hyperstructures; and finally that the result of this process of ecological evolution is not one new eukaryote, but many new eukaryote species. In sum, our ecology-first theory of the origin of life requires that integration of ecologies leads to new ecologies rather than individuals. What is striking about all life is not that it is made up of individuals, but that all individuals that are alive are part of an integrated network of interactions.

While we have provided detailed evidence for how structural and motile hyperstructures may have evolved, and provided a number of unique predictions, clearly this hypothesis will require significant development in the future. One of its benefits, in the meantime, is that it integrates many apparently separate problems of the origins of a variety of eukaryotic properties into a single process and does so based on principles that we have already demonstrated to be at work at previous levels of evolution.
